# Whole-Genome Doubling Affects Pre-miRNA Expression in Plants

**DOI:** 10.3390/plants10051004

**Published:** 2021-05-18

**Authors:** Salvatore Esposito, Riccardo Aversano, Pasquale Tripodi, Domenico Carputo

**Affiliations:** 1 CREA Research Centre for Cereal and Industrial Crops, 71122 Foggia, Italy; salvatore.esposito@crea.gov.it; 2 Department of Agricultural Sciences, University of Naples Federico II, 80055 Portici, Italy; riccardo.aversano@unina.it; 3 CREA Research Centre for Vegetable and Ornamental Crops, 84098 Pontecagnano, Italy; pasquale.tripodi@crea.gov.it

**Keywords:** autopolyploid, microRNA, *Solanum commersonii*, genomic shock, RNAseq

## Abstract

Whole-genome doubling (polyploidy) is common in angiosperms. Several studies have indicated that it is often associated with molecular, physiological, and phenotypic changes. Mounting evidence has pointed out that micro-RNAs (miRNAs) may have an important role in whole-genome doubling. However, an integrative approach that compares miRNA expression in polyploids is still lacking. Here, a re-analysis of already published RNAseq datasets was performed to identify microRNAs’ precursors (pre-miRNAs) in diploids (2x) and tetraploids (4x) of five species (*Arabidopsis thaliana* L., *Morus alba* L., *Brassica rapa* L.*,* *Isatis indigotica* Fort., and *Solanum commersonii* Dun). We found 3568 pre-miRNAs, three of which (pre-miR414, pre-miR5538, and pre-miR5141) were abundant in all 2x, and were absent/low in their 4x counterparts. They are predicted to target more than one mRNA transcript, many belonging to transcription factors (TFs), DNA repair mechanisms, and related to stress. Sixteen pre-miRNAs were found in common in all 2x and 4x. Among them, pre-miRNA482, pre-miRNA2916, and pre-miRNA167 changed their expression after polyploidization, being induced or repressed in 4x plants. Based on our results, a common ploidy-dependent response was triggered in all species under investigation, which involves DNA repair, ATP-synthesis, terpenoid biosynthesis, and several stress-responsive transcripts. In addition, an ad hoc pre-miRNA expression analysis carried out solely on 2x vs. 4x samples of *S. commersonii* indicated that ploidy-dependent pre-miRNAs seem to actively regulate the nucleotide metabolism, probably to cope with the increased requirement for DNA building blocks caused by the augmented DNA content. Overall, the results outline the critical role of microRNA-mediated responses following autopolyploidization in plants.

## 1. Introduction

Polyploidy is the term used to denote the presence of more than two complete chromosome sets in somatic cells, tissues, or individuals. It is quite rare in animals, whereas it is widespread in angiosperms [1]. Polyploid plants are traditionally distinguished as autopolyploids and allopolyploids. The former designate individuals formed from within one species by the doubling of structurally similar, homologous chromosome complements; the latter combine genomes from more than one species via hybridization and the subsequent doubling of differentiated, nonhomologous (homoeologous) chromosomes. Polyploidy causes several changes at the genetic, epigenetic, transcriptional, and metabolic network levels [2]. Although many aspects of the advantages of being polyploid have been elucidated over the last decades, the significance of whole-genome doubling (WGD) *per se* is still unclear. From this perspective, autopolyploids represent a par-excellence model to broaden our understanding of WGD in evolution and practical plant breeding. Notably, several studies on natural autopolyploids have led to the hypothesis that WGD can help to tolerate better eventual adverse conditions [3,4,5,6,7]. For example, tetraploid turnip [8], citrus [9], and black locust [10] coped with salt stress better than their respective 2x progenitors. Similarly, tetraploid *Brassica napus* and *Dendranthema nankingense* showed higher tolerance to salt and drought stress conditions [11,12].

However, the mechanisms behind these improved performances are still unclear. Most investigations are related to allopolyploids [13,14,15,16,17], whereas autopolyploids have received relatively scant research attention. Recent studies have provided evidence that micro-RNAs (miRNAs) may have an important role during the genomic shock associated with polyploid formation. MiRNAs are essential post-transcriptional gene regulators and have been reported to be involved in various plant growth stages, developmental transition, and the response to biotic and abiotic stresses [18,19]. They are a class of non-coding single-stranded RNA molecules with a length of approximately 21 nucleotides. MiRNAs usually originate from intergenic regions (i.e., non-protein-coding sequences), referred to as *MIR* genes (genes encoding miRNA). Like most mRNAs, *MIR* genes are transcribed by DNA-dependent RNA polymerase II (Pol II), and then modified (i.e., 5′ capped, 3′ polyadenylated, and/or spliced) to produce primary miRNAs (pri-miRNAs) [20,21]. These are then folded into hairpin-like structures consisting of a terminal loop, an upper stem (the miRNA/miRNA * region), a lower stem, and two arms. Pri-miRNAs can be recognized and cropped by Dicer-like RNase III endonucleases (DCLs) into 60–150 nucleotide (nt) stem-loop precursor miRNAs (pre-miRNAs). A second cleavage then occurs 20–24 nucleotides from the first cleavage site, releasing the miRNA/miRNA * (guide strand/passenger strand) duplex from the stem [22,23]. Once the miRNA/miRNA * duplex has been methylated by HUA Enhancer 1 (HEN1), plant homolog exportin-5/Exp5 (HST) and other unknown factors can export them to the cytoplasm. Here, one strand of the duplex (the passenger strand, miRNA *) is degraded, whereas the other one (the guide strand, miRNA) is incorporated into an RNA-induced silencing complex (RISC) containing the Argonaute (AGO) protein [24,25]. Most miRNAs load into AGO1-containing RISC to guide the post-transcriptional gene silencing (PTGS) of complementary mRNA [17,18,19]. Burgeoning evidence indicates that miRNAs may arise from *MIR* genes and transposable elements (TEs) localized into intergenic regions. This generates a feedback mechanism, where those TE-derived miRNAs affect the TE transcripts by sequence complementarity [26]. Due to their intimate relevance to miRNA synthesis, understanding pre-miRNA functions in the control of miRNAs’ biogenesis has recently become a key research question, as also reported by [27,28,29,30].

Among autopolyploid crops, the cultivated potato *Solanum tuberosum* (2n = 4x =48) is the most important worldwide in terms of cultivated area and consumption per capita. Breeding efforts to introgress novel traits into the cultivated form often require the exploitation of related tuber-bearing *Solanum* species; since most of them are diploid (2n = 2x = 24), chromosome doubling may be required to bypass sexual barriers [31]. To exploit the resistance genes of 2x *S. commersonii*, we produced synthetic autotetraploids (2n = 4x = 48) of this species. Developed materials proved to be useful not only for breeding but also for investigating the phenotypic and molecular effects of WGD. Our results revealed a perturbation of both transcriptome and metabolome and also highlighted a genotypic-dependent response [32]. Changes were associated with a phenomenon known as ‘nucleotide pool imbalance’, responsible for genomic instability events such as increased genetic recombination, chromosomal aberrations, and DNA breaks [32,33,34,35,36]. The question of what mechanism drives genes to be differentially expressed in 2x vs. 4x *S. commersonii*, but more generally in autopolyploids, is still open. The recent literature clearly shows that ploidy-associated differences are either species-specific or stochastic. However, evidence of common WGD responses for ecological adaptation is still lacking. An integrative approach that compares the molecular responses of several autopolyploids might help to answer some of the still unresolved questions. With this in mind, we performed a meta-analysis of pre-miRNAs in 2x and synthetic 4x genotypes, for which expression data are available in the literature. Our samples belonged to five different species, namely *Arabidopsis thaliana* L. [37], *Isatis indigotica* Fort. [38], *Brassica rapa* L. [39], *S. commersonii* Dun., and *Morus alba* L. [40]. *I. indigotica* (dyer’s woad), *B. rapa* L. (turnip), and *A. thaliana* are species of the Brassicaceae family, *M. alba* (white mulberry) is a member of the Moraceae family*,* and *S. commersonii* belongs to the *Solanaceae* family. All of the selected studies aimed to identify differentially expressed genes in synthetic 4x vs. 2x in different tissues, including leaves [39], sepals [36], and immature floral buds [38]. Since all 4x samples were obtained under similar conditions (anti-mitotic treatments), processed with similar technologies, and with no abiotic and/or biotic stresses imposed, we assumed that these data fit well for our purposes. Exploiting the comparative data generated, we attempted to bridge the gap of knowledge between WGD and transcriptomic and metabolomic changes experienced by synthetic *Solanum* autotetraploids.

## 2. Results

### 2.1. A Meta-Analysis in Different Plant Species Revealed Pre-miRNA Putatively Involved in WGD

Here, we performed a meta-analysis by combining data from different studies. However, since small RNAseq data (smRNAseq) on 2x vs. 4x were not available in the literature, we used a total of 600M already available RNAseq reads belonging to 2x and synthetic 4x genotypes of *A. thaliana* L.*, I. indigotica* Fort*., B. rapa* L*., S. commersonii* Dun*.,* and *M. alba* L. Even though we cannot exclude bias due to the typology of the experiments, we assumed that RNAseq data might help to identify pre-miRNAs, rather than mature miRNAs, for which specific small RNA libraries are required. Following a quality check, adapters removal, and filtering for low-quality sequences, more than 477M cleaned reads were further processed. Using this subset, 3568 pre-miRNAs were identified in all samples, with the lowest number (46) in *M. alba* and the highest (222) in *B. rapa* (Table 1).

**Figure 1:**
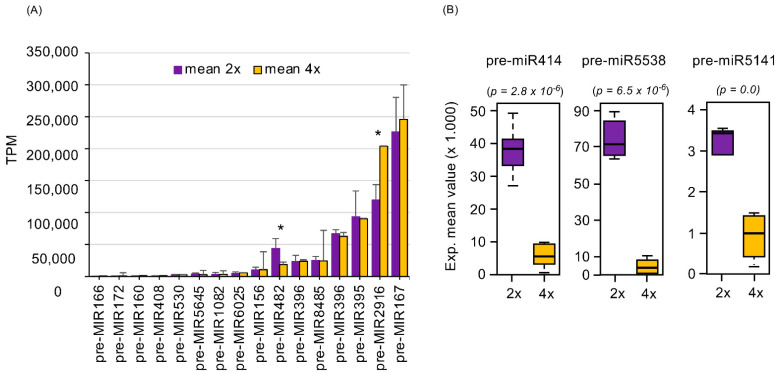
Expression profile of common pre-miRNAs identified in our study (**A**). Values are expressed in transcript per million (TPM) and as the mean of 2x (purple) and 4x (orange) genotypes. Standard error values are also reported. Box plot of expression mean value was calculated as transcript per million (TPM) of pre-miR414, pre-miR5538, and pre-miR5141 (**B**). Significant statistical differences (*p* < 0.05) are marked with asterisk.

When diploids and their respective tetraploids within each species were compared, 2x- and 4x-specific pre-miRNAs were found (Table S1). The highest number of 2x-specific pre-miRNAs was observed in *B. rapa* (67), whereas the lowest was in *M. alba* (2). Likewise, *S. commersonii* presented a number of 4x-specific pre-miRNAs, higher than *B. rapa* and *M. alba* (46, 4 and 6, respectively). Higher similarities were observed among the *Brassicaceae* species vs. *Solanaceae* and *Moraceae*. For example, among the pre-miRNAs identified in all diploids, 14 were homologues between *A. thaliana* and *B. rapa,* and 20 between *B. rapa* and *I. indigotica* (Figure S1A). A similar pattern was also observed among all 4x (Figure S1B). Indeed, 12 pre-miRNAs were found in both *A. thaliana* and *B. rapa,* and 8 in *B. rapa* and *I. indigotica.* By contrast, most of the pre-miRNAs identified in *S. commersonii* and *M. alba* were species-specific. The 2x and 4x of all species were also compared (Figure 1A). Among all pre-miRNAs identified, 19 homologous loci were found within 2x, whereas 16 were common to all 4x. As shown in Figure 1A, 13 out of 16 homology loci displayed a similar expression behavior in both 2x and 4x. By contrast, differences in the expression levels of pre-miRNA482b, pre-miRNA2916, and pre-miRNA167b were evident. Indeed, following polyploidization, pre-miRNA482 decreased its expression (from 44.099 TPM on average in 2x to 18.592 TPM in 4x, *p* value = 0.05), whereas both pre-miRNA2916 and pre-miRNA167b increased their expression (from 119.929 TPM to 204.107 TPM, *p* value = 0.05 and from 226.726 TPM to 245.764 TPM, *p* value = 0.4, respectively) (Figure 1A). Three pre-miRNAs (pre-miR414, pre-miR5538, and pre-miR5141) were expressed in all 2x, but not in all 4x (Figure 1B and Figure S2). Pre-miR414 was not expressed in *M. alba* or *B. rapa,* whereas pre-miR5538 and pre-miR5141 were lacking in *I. indigotica.* Using the mean of the TPM values in the remaining samples, a significant reduction was found for pre-miR414 (*p* value < 0.05), pre-miR5538 (*p* value < 0.05) and pre-miR5141 (*p* value = 0) (Figure 1B). Targets for these pre-miRNAs were predicted and further analyzed in the next paragraph.

### 2.2. Target Prediction and GO Enrichment Analysis Clarify the Function of Selected Pre-miRNAs

The effects of such deregulation following WGD may impair the expression of the miRNA-complementary mRNA sequences. Therefore, targets for pre-miR414, pre-miR5538, and pre-miR5141were predicted, and their functions annotated as follows: pre-miR414 had 202 targets, including a dozen transcripts annotated as transcription factors (TFs), whereas pre-miR5538 was a regulator of the expression of 88 different mRNA sequences, most of which with unknown functions. Forty targets were predicted for pre-miR5141; they are involved in several processes, including ATP synthesis, transporter, and oxidoreductase activities. Interestingly, these three pre-miRNAs target more than 50 transposable elements (TEs), suggesting a possible role in silencing TEs during the normal 2x condition. The iTAK pipeline was also implemented to identify regulators within the miRNA targets. In total, we found 19 TFs and 15 transcriptional regulators (TRs) (Table 2). Among them, we annotated five plant-specific TFs belonging to the *B3* superfamily, which encompasses well-characterized families (e.g., the Auxin Response Factor (*ARF*) and the *LAV* families); three trihelix TFs, a newly discovered family with functions in responses to environmental stresses and late embryogenesis regulation; and two *MYB*-related TFs, critical factors in regulatory networks controlling development, metabolism, and responses to stresses. We also found 15 TRs; among them, the *Sucrose Non-Fermenting 2* (*SNF2*) transcriptional regulators, a family of helicase-like proteins that direct energy derived from ATP hydrolysis into the mechanical remodeling of the chromatin structure, were the most abundant (Table 2). Enrichment analysis of GO annotation allowed us to identify the functions of pre-miRNAs’ targets; a semantic TreeMap was obtained to summarize their GO categories (Figure S3). Starting from pre-miR414 (Figure S3A), we observed high values for the category involved in DNA repair (GO:0006281). This category includes GO terms with functions in the cellular response to stimulus (GO:0051716), cellular response to DNA damage stimulus (GO:0006974), and cellular response to stress (GO:0033554). GO categories with function in developmental processes (GO:0032502), reproduction (GO:0000003), and regulation of biological processes (GO:0050789) were also enriched, confirming that pre-miR414 targets participate in several important plant functions including growth, metabolism, physiological and morphological adjustments, and defense strategies. As for pre-miR5538 (Figure S3B), only three GO enriched terms were identified: transporter activity (GO:0005215), hydrolase activity involving O-compounds (GO:0004553), and oxidoreductase activity (GO:0016491). This enrichment is due to the presence of *S-isoprenylcysteine O-methyltransferase*, an enzyme involved in terpenoid backbone biosynthesis that may probably influence the production of secondary metabolites. Three GO-enriched terms identified as transferase activity (GO:0016740), protein binding (GO:0005515), and catalytic activity (GO:0003824) were enriched among pre-miR5141 targets (Figure S3C). As last step, we investigated the transcriptome data available for the leaves, flowers, roots, and fruits of *A. thaliana* (Figure S4). This allowed us to verify if the predicted targets were widely expressed in different tissues, and thus to exclude any bias due to the samples used in each experiment. The heat map-based expression profiles revealed a dynamic expression of all targets across tissues, although no tissue-specificity was observed (Figure S4).

### 2.3. 2x vs. 4x Whole-MicroRNA Expression Analysis in Tuber-Bearing S. Commersonii Allowed the Identification of Polyploidy-Associated Transcriptional Regulators

**Figure 2:**
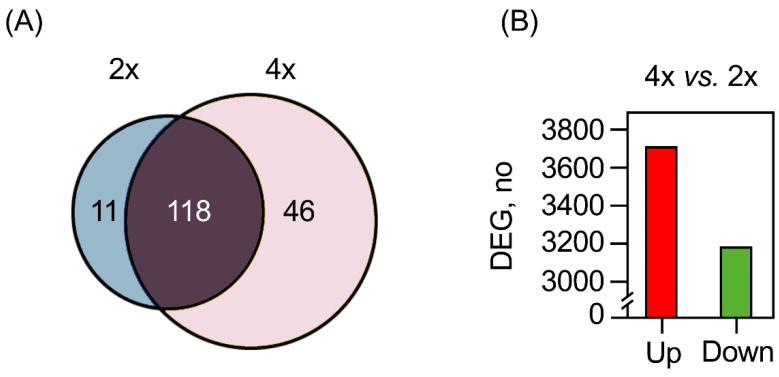
(**A**) Venn diagram showing the number of common and unique pre-miRNAs identified in 2x and 4x *S. commersonii*. (**B**) Number of differentially expressed genes (DEGs) obtained comparing 4x *S. commersonii* with its 2x progenitor.

Exploiting the comparative data generated, we attempted to bridge the gap of knowledge between WGD and transcriptomic and metabolomic changes experienced by synthetic *Solanum* autotetraploids [32]. Whether or not such changes were the consequences of microRNA dysregulation remains elusive. For this reason, more than 215M reads were produced for both cmm1T (2x) and cmm30 (4x), with an average of 33M reads from each sample (data not shown). Following the filtering step, we identified 129 (in cmm1T) and 164 (in cmm30) known pre-miRNAs, corresponding to 82 and 97 conserved families available in miRbase, respectively. Among all identified pre-miRNAs, 11 were 2x-specific, since they were found uniquely expressed in cmm1T; 46 were 4x-specific, as expressed only in cmm30; and 118 were in common to both ploidies (Figure 2A).

Among the 2x-specific pre-miRNAs, we identified pre-miR167d, pre-miR414, pre-miR5538, pre-miR6236, and pre-miR6240. Among those classified as 4x-specific, we found four members belonging to the pre-miR8024 family (a, b, c, and d), three members belonging to pre-miR169 (b, d, and g), two members belonging to pre-miR482 (a and b), and pre-miR167b. The potential miRNA targets were predicted from the *S. commersonii* transcriptome. Regarding the 4x-specific pre-miRNAs, 5177 potential targets were found; in contrast, 2571 potential targets were identified for the 2x-specific miRNA (data not shown). No significant GO terms were detected for either subset. Hierarchical clustering analysis of 118 common pre-miRNAs allowed for the identification of two different sub-groups: 68 increased pre-miRNAs in the 4x compared to the 2x and 50 decreased in the 4x (Figure S5). For the 68 pre-miRNAs scored as more abundant in the tetraploid form, 4449 potential targets were predicted; they were assigned to GO terms under the 3 GO domains (biological process, cellular components and molecular function). Most of the predicted targets were annotated to be involved in the “response to stress and defense response” and the “symplast and cell–cell junctions” (Figure S6). Regarding the 50 decreased pre-miRNAs in 4x cmm30, 3711 potential targets were identified, and were mostly classified into the “production of small RNA molecules” and the “regulation of gene expression through epigenetic” GO terms (Figure S7). Overall, the GO annotation of the genes targeted by ploidy-affected miRNAs provided evidence that the most commonly recurring GO term was “nucleotide and purine bindings” in both the induced and repressed datasets.

Since such changes in pre-miRNA expression affect the behavior of their targets, we performed a differential analysis of *S. commersonii* at the gene level. Following polyploidization, 6843 differentially expressed genes (DEGs) were identified; 3661 (54%) of them were up-regulated, while 3182 (46%) were down-regulated (Figure 2B). Among the induced genes, 714 were annotated as targets of less abundant pre-miRNAs. Eight were annotated as TFs belonging to *AP2/ERF*, *MYB*, *Zinc-finger*, *C2H2,* and *WRKY* families; all showed log_2_FC values ranging from four- to sixfold compared to the 2x progenitor. In contrast, 805 down-regulated genes were identified as targets of the most abundant pre-miRNAs.

## 3. Discussion

Although several transcriptomic studies have been performed to study the effects of WGD and some relevant genes have been associated with this phenomenon [32,33,34,35,36], a number of molecular and biological aspects are still unknown. It has been hypothesized that post-transcriptional regulation mediated by small RNAs may play a pivotal role in regulating gene expression following genome doubling. Here, we showed that autopolyploidization might induce changes in pre-miRNA expression, which triggers alterations in the transcriptional activity of several target genes, mainly involved in DNA metabolism, secondary metabolism, and stress tolerance. To compare our results with those found in the literature, hereafter, we refer to pre-miRNAs as miRNAs.

### 3.1. Meta-Analysis of miRNAs Provides New Insight into The Biological Processes of Autotetraploid Formation

Using independent synthetic tetraploids of different plant species, we were able to address whether transcriptomic perturbations due to the presence of miRNA precursors occurred. Although 16 homologue miRNAs were found in all 2x and 4x, two of them (miR482 and miR2916) significantly changed their expression, being either induced or repressed in 4x vs. 2x. MiR482 is known to be active in response to the inoculum of viruses, *Verticillium dahliae*, soybean cyst nematodes, and *Ralstonia solanacearum* [41,42,43,44,45,46,47,48]. In particular, it is a negative regulator of tolerance to pathogens by targeting conserved sequences encoding the P-loop of NBS–LRR resistance proteins. As far as miR2916 is concerned, it has been reported to be responsive to boron stress in barley [49] and related to salinity tolerance in rice [50]. However, its role has not been investigated yet. In addition, we also found three different conserved miRNAs (miR414, miR5538, and miR5141) that were mainly active in 2x but not in all 4x. MiR414 expression was reduced after WGD, as recently reported in newly formed autotetraploids of *C. nankingense* [42]. Interestingly, we found that miR414 primarily targets transcriptional regulators belonging to *MYB*, *B3*, *AP2/ERF,* and *bZIP* families. All of them were described as playing a key role in plant growth and development, physiological adjustments and morphological changes, secondary metabolism, and defense against stressors [44,45]. Since little is known about the transcriptional regulators active during polyploidization, the transcription factors identified in this survey represent promising candidates for being experimentally validated. The activity of miR414 has been also associated with the regulation of the carotenoid/apocarotenoid biosynthetic pathway [46] and the auxin and energy metabolisms [47]. These results highlight miR414 as a potential candidate in the regulation of changes triggered by WGD. As far as miR5538 is concerned, we found that one of its target genes is *protein-S-isoprenylcysteine O-methyltransferase*, which is involved in the terpenoid backbone biosynthesis. The enzyme catalyzes the post-translational methylation of isoprenylated C-terminal cysteine residues [48]. This is particularly interesting since it has been long recognized that polyploidization can change the quality and quantity of secondary metabolites. Recently, Gaynor et al. [49] conducted a meta-analysis aimed at comparing the composition and concentration of secondary metabolites among closely related plant species differing in ploidy level. Although the authors suggested that it was not possible to determine the overall effect of whole-genome doubling on secondary metabolites, mainly due to the heterogeneity in methodological and analytical approaches used, targeted studies proved their increase in many plant species. For example, in induced polyploids of *Citrus limon* L. [50], *Cannabis sativa* [51], *Thymus vulgaris* L. [52], and *Lippia integrifolia* [53], increased terpene levels and elevated essential oil concentration were found. Many terpenoids have biological activities, and, in higher plants, diterpenes and sesquiterpenes are synthesized as a part of plant defense mechanisms [54,55]. Given that, the action of ploidy-dependent microRNA, such as miR5538, may trigger the changes in the chemical composition experienced by several autopolyploids, which, in turn, could confer a benefit in terms of greater fitness and thereby provide an important factor in their success. The last miRNA that we mainly found mostly expressed at the 2x level was miR5141. It targets ATP synthase, which is involved in ATP synthesis and ATP utilization [56]. Autopolyploids usually exhibit phenotypic and physiological divergences when compared with their 2x counterparts (i.e., in the size of plant organs, stress response, and flowering time). From a biochemical standpoint, such changes represent an energy-consuming process, and this might explain the role of miR5141 in the energy metabolism linked to WGD. Taken as a whole, our data indicated that in all five species investigated, WGD triggered transcriptional changes in pre-miRNAs that target genes mainly involved in stress tolerance, energy, and secondary metabolism. It is tempting to speculate that such a coordinated ploidy-dependent network as we observed might potentiate the evolutionary adaptation of polyploids, particularly under unstable, stressful environments, facilitating cellular states neither typically found in nor well tolerated by diploids. However, further experiments are needed to bolster this hypothesis from an evolutionary perspective.

### 3.2. Genome Doubling in S. Commersonii Affects miRNAs Targeting Purine Nucleotides

Previous studies reported that *S. commersonii* autopolyploidization alters metabolite content [57], methylation patterns [58], and transcriptome activity [32]. Whether or not such changes were the consequences of microRNA dysregulation remains elusive. Our study found 129 pre-miRNAs in 2x *S. commersonii* and 164 in its related 4x; 11 of them were 2x-specific, 46 4x-specific and 118 were commonly expressed between the ploidies. As far as commonly expressed miRNAs are concerned, 68 increased their expression, and 50 decreased. GO annotation of their target genes revealed that “nucleotide and purine bindings” were highly enriched in both differentially expressed groups. It is known that purine nucleotides participate in many biochemical processes in plants. They are building blocks for nucleic acid synthesis, an energy source, and precursors for the synthesis of primary and secondary metabolites [59]. Fasano et al. [32] reported a depletion of adenosine, guanine, and guanosine in newly synthesized autotetraploids of *S. commersonii*. The authors hypothesized a possible imbalance of the nucleotide pool due to an impairment of nuclear metabolites (especially those involved in DNA synthesis) to cope with the increased requirement for DNA building blocks caused by the augmented DNA content. In particular, intracellular dNTP pool sizes are known to be tightly regulated, and their imbalance has genotoxic consequences [59,60]. A retrospective view of the research on the genomic changes triggered by WGD confers reliability to the “nucleotide pool imbalance” hypothesis. In this scenario, the group of ploidy-dependent miRNA we identified may represent the missing link between WGD and transcriptomic and metabolomic changed described by Fasano et al. [32]. Finally, they represent promising candidates for further research.

## 4. Materials and Methods

### 4.1. Plant Material, RNA Extraction and Illumina Sequencing

The synthetic autotetraploid of *S. commersonii* was generated as previously described by [51]. In detail, a clone of diploid (2n = 2x = 24) *S. commersonii* (PI 243503), coded cmm1T, was subjected to oryzalin treatment, and the derived tetraploid (2n = 4x = 48) was coded cmm30. Clonal progenies were stabilized before being used in this study. All plants were also maintained and propagated in vitro on Murashige and Skoog (MS) medium [61], including salts, vitamins, sucrose (30 g L^−1^), and microagar (9 g L^−1^) and adjusted to pH 5.8. The cultures were maintained in a walk-in growth chamber at 25 °C with a 16 h:18 h (light:dark) photoperiod at 125 L mol m^2^ s^−1^ irradiance provided by a cool white fluorescent tube (TL-D 58W/33-640 1SL; Philips, Eindhoven, the Netherlands). To collect material for molecular analyses, six healthy and uniform plants of both 2x cmm1T and 4x cmm30 were grown in 25-cm-diameter pots (the spacing between the plants was 30 cm) in a temperature-controlled glasshouse (24 °C:16 °C, day:night, relative humidity 65–75%, and natural photoperiod). For each sample, total RNA was isolated from the leaf tissues using a Spectrum Plant Total RNA kit (Sigma-Aldrich, St. Louis, MO, USA) according to the manufacturer’s protocol. RNA concentrations were determined using a NanoDrop ND-1000 spectrophotometer (Thermo Scientific, Wilmington, NC, USA) and its integrity was verified using a bioanalyzer (Agilent Technologies, Santa Clara, CA, USA). Three μg of total RNA from each sample was sent to the UMN Genomic Center (University of Minnesota, USA) for library preparations. Six cDNA libraries (three biological replicates from the leaves of the 2x control and three from the 4x) were subsequently prepared for RNA-seq experiments with the Illumina HiSeq 2500 sequencing platform, providing 125 bp paired-end reads for a total of 30M reads/samples. The quality assessment was carried out using FASTQC tool version 0.10.0 (http://www.bioinformatics.babraham.ac.uk/projects/fastqc, accessed on 10 December 2020), whereas trimming was performed with Trimmomatic-0.3330 [62].

### 4.2. Pre-miRNA Identification

To increase the number of analyzed samples, RNAseq data corresponding to 18 genotypes (nine 2x and their respective 4x) of five different plant species (*A. thaliana*, *S. commersonii*, *M. alba*, *B. rapa,* and *I. indigotica*) (Table 1) were downloaded from NCBI (https://www.ncbi.nlm.nih.gov/sra, accessed on 10 November 2019) using sratoolkit (http://ncbi.github.io/sra-tools/, accessed on 10 November 2019). Similar to the cmm1T RNAseq data, the quality assessment was carried out using FASTQC tool version 0.10.0 (http://www.bioinformatics.babraham.ac.uk/projects/fastqc, accessed on 10 December 2020), whereas trimming was performed with Trimmomatic-0.3330 [62]. Cleaned reads were mapped against the repository of miRNA precursors (http://www.mirbase.org/ftp.shtml, accessed on 17 December 2020) using Kallisto with default parameters [63]. Pre-miRNA names were automatically assigned based on the identity within the miRbase repository. Redundant sequences (i.e., the same pre-miRNA identified in different species) were removed. Pre-miRNAs with abundance equal to 0 were discarded, whereas the remaining were kept for further analysis. The mean of the transcript per million (TPM) values was used for each pre-miRNA to normalize data across samples. The selected pre-miRNAs were tested for statistically significant effects using a *t*-test (*p* ≤ 0.05).

### 4.3. Prediction of Potential miRNA Targets and GO Enrichment

To predict the mRNA targets of putative miRNAs, the psRNATarget tool (http://plantgrn.noble.org/psRNATarget/, accessed on 19 February 2021) was used. Pre-miRNAs sequences identified in our study were mapped against the *A. thaliana* transcripts (version 10, TAIR) with default parameters. GO terms (http://www.Geneontology.org/, accessed on 19 February 2021) and the Kyoto Encyclopedia of Genes and Genomes (http://www.Genome.jp/kegg/, accessed on 19 February 2021) were used to investigate the function of target genes further. In detail, GO enrichment analysis was performed using AgriGO [62] with the following parameters: hypergeometric statistical test method, multitest adjustment Hockberg FDR < 0.05, and three as the minimum number of mapping entries. Significant values were sorted by enrichment score, and GO redundancy was removed with the REVIGO tool [63]. Target sequences were also used to identify and classify transcription factors (TFs) with iTAK (Plant Transcription factor & Protein Kinase Identifier and Classifier; http://bioinfo.bti.cornell.edu/cgi-bin/itak/index.cgi, accessed on 15 March 2021). Finally, the transcriptional activity of target sequences was estimated using the publicly available RNAseq data of *A. thaliana* deposited in the EMBL-EBI Gene Expression Atlas (https://www.ebi.ac.uk/gxa/plant/experiments, accessed on 15 March 2021). In detail, the FPKM values of four tissues (leaves, roots, flowers, and fruits) were downloaded and used as an input to drown heat maps.

### 4.4. Identification of Differentially Expressed Genes and Transposable Elements

The same libraries used for pre-miRNA identification were loaded in the artificial intelligence RNAseq (AIR) online tool (https://transcriptomics.cloud, accessed on 10 December 2020), and a new RNA-seq experiment was chosen. Gene expression levels were calculated using the geometric normalization and per-condition dispersion method by quantifying the Illumina reads according to the FPKM (fragments per kilobase per million mapped fragments). Fold-changes were reported as the log (base 2) of the normalized read count abundance for the 4x samples divided by the read count abundance of the 2x samples.

## 5. Conclusions

The present work reports a meta-analysis approach for the investigation of pre-miRNAs involved in plant autopolyploidization. This represents the first attempt toward this objective, shedding light on some aspects of the complicated functioning of regulatory networks in autopolyploids. Overall, we found a subset of shared pre-miRNA putatively involved in WGD. They may be responsible for changes in DNA repair mechanisms, secondary metabolism, and stress tolerance. In addition, the ad hoc pre-miRNA expression analysis carried out solely on 2x vs. 4x *S. commersonii* samples suggested that the ploidy-dependent miRNAs identified might regulate the nucleotide metabolism, paving new paths towards a deeper understanding of the model proposed by Fasano et al. [32] to explain the transcriptomic and metabolomic changes experienced by synthetic *Solanum* autotetraploids. To validate the results presented here, further studies are on-going. For this purpose, transgenic plants defective in our pre-miRNA candidates will be produced. In addition, we are willing to share data and plant materials to anyone interested in further studies aimed at elucidating these aspects further.

## Figures and Tables

**Table 1:** Number of identified pre-miRNAs. Tissues used in each experiment along with ploidy level are also reported.

SRA Code	Species	Ploidy	Tissues	Pre-miRNA Identified, No.	Reference
SRR2084347	*M. alba* L.	2x	leaves	47	[40]
SRR2084348	*M. alba* L.	2x	leaves	57	
SRR2084349	*M. alba* L.	2x	leaves	51	
SRR6927975	*A. thaliana* L.	2x	sepals	160	[37]
SRR6927977	*A. thaliana* L.	2x	sepals	179	
SRR6927978	*A. thaliana* L.	2x	sepals	173	
SRR1565773	*I. indigotica* Fort.	2x	leaves	110	[38]
SRR1565774	*I. indigotica* Fort.	2x	leaves	116	
SRR1565775	*I. indigotica* Fort.	2x	leaves	99	
SRR5451141	*B. rapa* L.	2x	floral buds	222	[39]
This study	*S. commersonii* Dun.	2x	leaves	187	Our study
This study	*S. commersonii* Dun.	2x	leaves	185	
This study	*S. commersonii* Dun.	2x	leaves	194	
SRR2084350	*M. alba* L.	4x	leaves	63	[40]
SRR2084351	*M. alba* L.	4x	leaves	49	
SRR2084352	*M. alba* L.	4x	leaves	46	
SRR6927976	*A. thaliana* L.	4x	sepals	168	[37]
SRR6927981	*A. thaliana* L.	4x	sepals	175	
SRR6927982	*A. thaliana* L.	4x	sepals	183	
SRR1565776	*I. indigotica* Fort.	4x	leaves	99	[38]
SRR1565777	*I. indigotica* Fort.	4x	leaves	112	
SRR1565778	*I. indigotica* Fort.	4x	leaves	114	
SRR5451140	*B. rapa* L.	4x	floral buds	196	[39]
This study	*S. commersonii* Dun.	4x	leaves	192	Our study
This study	*S. commersonii* Dun.	4x	leaves	201	
This study	*S. commersonii* Dun.	4x	leaves	190	

**Table 2:** List of transcription factors (TF) and transcriptional regulators (TR) identified among the pre-miRNA targets. The number of transcripts/family is also reported.

Family	Type	Transcripts, No.
B3	TF	5
Trihelix	TF	3
Alfin-like	TF	2
RWP-RK	TF	2
MYB-related	TF	2
C2H2	TF	2
SBP	TF	1
C3H	TF	1
WRKY	TF	1
SNF2	TR	3
PHD	TR	2
Others	TR	2
SET	TR	1
Jumonji	TR	1
AUX/IAA	TR	1
GNAT	TR	1
SWI/SNF-SWI3	TR	1
SWI/SNF-BAF60b	TR	1
HMG	TR	1
mTERF	TR	1

## Data Availability

Bioproject ID: PRJNA647649.

## Supplementary Materials

The following are available online at https://www.mdpi.com/article/10.3390/plants10051004/s1. Figure S1. Venn diagrams showing the number of pre-miRNAs identified in all 2x (A) and 4x (B). Figure S2. Histograms showing the expression pattern for miR414, miR5538, and miR5141 in the five species under study. Values are expressed in transcript per million (TPM). Figure S3. REVIGO tree map summarizing the enriched GO terms for miR414 (A), miR5538 (B), and miR5141(C). Similar colors denote semantic similarity, and each rectangle is proportional to the relative enrichment of the GO term as compared to the whole genome. Figure S4. Heatmap showing the expression pattern of targets for pre-miR414 (A), pre-miR5538 (B), and pre-miR5141(C) in four tissues of *A. thaliana* (leaves, flowers, roots, and fruits). The color scale indicates FPKM (fragments per kilobase per million sequenced reads) expression values. Figure S5. HeatMap showing the expression pattern of 118 common pre-miRNAs identified in *S. commersonii*. Increased and decreased pre-miRNAs in the 4x as compared to the 2x are shown. Figure S6. Hierarchical clustering showing the enriched GO terms for the biological process (A), cellular component (B), and molecular function (C) identified among over-expressed pre-miRNAs in *S. commersonii*. The significant terms (adjusted *p* ≤ 0.05) are marked with color, while non-significant terms are shown as white boxes. The degree of color saturation of a box is positively correlated with the enrichment level of that term. Figure S7. Hierarchical clustering showing the enriched GO terms for the biological process (A), cellular component (B), and molecular function (C) identified among down-expressed pre-miRNAs in *S. commersonii*. The significant terms (adjusted *p* ≤ 0.05) are marked with color, while non-significant terms are shown as white boxes. The degree of color saturation of a box is positively correlated with the enrichment level of that term.

## References

[1] Mable B.K. (2004). Why polyploidy is rarer in animals than in plants: Myths and mechanisms. Biol. J. Linn. Soc..

[2] Lavania U.C., Srivastava S., Lavania S., Basu S., Misra N.K., Mukai Y. (2012). Autopolyploidy differentially influences body size in plants, but facilitates enhanced accumulation of secondary metabolites, causing increased cytosine methylation. Plant J..

[3] Soltis D.E., Albert V.A., Leebens-Mack J., Bell C.D., Paterson A.H., Zheng C., Sankoff D., Depamphilis C.W., Wall P.K., Soltis P.S. (2009). Polyploidy and angiosperm diversification. Am. J. Bot..

[4] Flagel L.E., Wendel J.F. (2009). Gene duplication and evolutionary novelty in plants. New Phytol..

[5] Kondrashov F.A. (2012). Gene duplication as a mechanism of genomic adaptation to a changing environment. Proc. R. Soc. B Biol. Sci..

[6] Crawford D.J., Doyle J.J., Soltis D.E., Soltis P.S., Wendel J.F. (2014). Contemporary and future studies in plant speciation, morphological/floral evolution and polyploidy: Honoring the scientific contributions of Leslie, D. Gottlieb to plant evolutionary biology. Philos. Trans. R. Soc. B Biol. Sci..

[7] Fisher K.J., Buskirk S.W., Vignogna R.C., Marad D.A., Lang G.I. (2018). Adaptive genome duplication affects patterns of molecular evolution in *Saccharomyces cerevisiae*. PLoS Genet..

[8] Meng H.-B., Jiang S.-S., Hua S.-J., Lin X.-Y., Li Y.-L., Guo W.-L., Jiang L.X. (2011). Comparison between a tetraploid turnip and its diploid progenitor (*Brassica rapa* L.): The adaptation to salinity stress. ASC.

[9] Saleh B., Allario T., Dambier D., Ollitrault P., Morillon R. (2008). Tetraploid citrus rootstocks are more tolerant to salt stress than diploid. Comptes Rendus Biol..

[10] Wang Z., Wang M., Liu L., Meng F. (2013). Physiological and proteomic responses of diploid and tetraploid black locust (*Robinia pseudoacacia* L.) subjected to salt stress. Int. J. Mol. Sci..

[11] Liu S.Y., Chen S.M., Chen Y., Guan Z.Y., Yin D.M., Chen F.D. (2011). In vitro induced tetraploid of *Dendranthema nankingense* (Nakai) Tzvel. shows an improved level of abiotic stress tolerance. Sci. Hortic..

[12] Xu Y., Zhong L., Wu X., Fang X., Wang J. (2009). Rapid alterations of gene expression and cytosine methylation in newly synthesized *Brassica napus* allopolyploids. Planta.

[13] Liu Y., Wang J., Ge W., Wang Z., Li Y., Yang N., Sun S., Zhang L., Wang X. (2017). Two highly similar poplar paleo-subgenomes suggest an autotetraploid ancestor of Salicaceae plants. Front. Plant Sci..

[14] Martinez Palacios P., Jacquemot M.P., Tapie M., Rousselet A., Diop M., Remoué C., Falque M., Lloyd A., Jenczewski E., Lassalle G. (2019). Assessing the response of small RNA populations to allopolyploidy using resynthesized *Brassica napus* allotetraploids. Mol. Biol. Evol..

[15] Chen Z.J., Sreedasyam A., Ando A., Song Q., De Santiago L.M., Hulse-Kemp A.M., Ding M., Ye W., Kirkbride R.C., Jenkins J. (2020). Genomic diversifications of five Gossypium allopolyploid species and their impact on cotton improvement. Nat Genet.

[16] Esposito S., Aversano R., Bradeen J.M., Di Matteo A., Villano C., Carputo D. (2018). Deep-sequencing of *Solanum commersonii* small RNA libraries reveals riboregulators involved in cold stress response. Plant Biol..

[17] Ma X., Wiedmer J., Palma-Guerrero J. (2020). Small RNA Bidirectional Crosstalk During the Interaction Between Wheat and *Zymoseptoria tritici*. Front. Plant Sci..

[18] Brant E.J., Budak H. (2018). Plant Small Non-coding RNAs and Their Roles in Biotic Stresses. Front. Plant Sci..

[19] Budak H., Akpinar B.A. (2015). Plant miRNAs: Biogenesis, organization and origins. Funct. Integr. Genom..

[20] Rogers K., Chen X. (2013). Biogenesis, turnover, and mode of action of plant microRNAs. Plant Cell.

[21] Rogers K., Chen X. (2012). MicroRNA biogenesis and turnover in plants. Quant. Biol..

[22] Voinnet O. (2009). Origin, biogenesis, and activity of plant microRNAs. Cell.

[23] Axtell M.J., Westholm J.O., Lai E.C. (2011). Vive la difference: Biogenesis and evolution of microRNAs in plants and animals. Genome Biol..

[24] Roberts J.T., Cardin S.E., Borchert G.M. (2014). Burgeoning evidence indicates that microRNAs were initially formed from transposable element sequences. Mob. Genet. Elem..

[25] Cho J. (2018). Transposon-Derived Non-coding RNAs and Their Function in Plants. Front. Plant Sci..

[26] Esposito S., Aversano R., D’amelia V., Villano C., Alioto D., Mirouze M., Carputo D. (2018). *Dicer-like* and *RNA-dependent RNA polymerase* gene family identification and annotation in the cultivated *Solanum tuberosum* and its wild relative *S. commersonii*. Planta.

[27] Ma Y., Yu Z., Han G., Li J., Anh V. (2018). Identification of pre-microRNAs by characterizing their sequence order evolution information and secondary structure graphs. BMC Bioinform..

[28] Fu X., Zhu W., Cai L., Liao B., Peng L., Chen Y., Yang J. (2019). Improved Pre-miRNAs Identification Through Mutual Information of Pre-miRNA Sequences and Structures. Front. Genet..

[29] Carputo D., Aversano R., Mason A.S. (2016). Potato breeding through chromosome manipulation. Polyploidy and Interspecific Hybridization for Crop Improvement.

[30] Fasano C., Diretto G., Aversano R., D’Agostino N., Di Matteo A., Frusciante L., Giuliano G., Carputo D. (2016). Transcriptome and metabolome of synthetic Solanum autotetraploids reveal key genomic stress events following polyploidization. New Phytol..

[31] Kaeppler S.M., Phillips R.L., Olhoft P. (1998). Molecular basis of heritable tissue culture-induced variation in plants. Somaclonal variation and Induced Mutations in Crop Improvement.

[32] Mathews C.K. (2006). DNA precursor metabolism and genomic stability. FASEB J..

[33] Tan E.H., Comai L., Henry I.M. (2016). Chromosome dosage analysis in plants using whole genome sequencing. Bio Protoc..

[34] Zwaenepoel A., Li Z., Lohaus R., Van de Peer Y. (2019). Finding evidence for whole genome duplications: A reappraisal. Mol. Plant.

[35] Robinson D.O., Coate J.E., Singh A., Hong L., Bush M., Doyle J.J., Roeder A.H.K. (2018). Ploidy and Size at Multiple Scales in the *Arabidopsis* Sepal. Plant Cell.

[36] Zhou Y., Kang L., Liao S., Pan Q., Ge X., Li Z. (2015). Transcriptomic Analysis Reveals Differential Gene Expressions for Cell Growth and Functional Secondary Metabolites in Induced Autotetraploid of Chinese Woad (*Isatis indigotica* Fort.). PLoS ONE.

[37] Braynen J., Yang Y., Wei F., Cao G., Shi G., Tian B., Zhang X., Jia H., Wei X., Wei Z. (2017). Transcriptome Analysis of Floral Buds Deciphered an Irregular Course of Meiosis in Polyploid *Brassica rapa*. Front. Plant Sci..

[38] Dai F., Wang Z., Luo G., Tang C. (2015). Phenotypic and Transcriptomic Analyses of Autotetraploid and Diploid Mulberry (*Morus alba* L.). Int. J. Mol. Sci..

[39] Feng J., Liu S., Wang M., Lang Q., Jin C. (2014). Identification of microRNAs and their targets in tomato infected with Cucumber mosaic virus based on deep sequencing. Planta.

[40] Tsushima D., Adkar-Purushothama C.R., Taneda A., Sano T. (2015). Changes in relative expression levels of viroid-specific small RNAs and microRNAs in tomato plants infected with severe and mild symptom-inducing isolates of Potato spindle tuber viroid. J. Gen. Plant Pathol..

[41] Dong B., Wang H., Song A., Liu T., Chen Y., Fang W., Chen S., Chen F., Guan Z., Jiang J. (2016). miRNAs Are involved in determining the improved vigor of autotetrapoid *Chrysanthemum nankingense*. Front. Plant Sci..

[42] Isah T. (2019). Stress and defense responses in plant secondary metabolites production. Biol. Res..

[43] Meraj T.A., Fu J., Raza M.A., Zhu C., Shen Q., Xu D., Wang Q. (2020). Transcriptional factors regulate plant stress responses through mediating secondary metabolism. Genes.

[44] Zinati Z., Shamloo-dashtpagerdi R., Behpouri A. (2016). In silico identification of miRNAs and their target genes and analysis of gene co-expression network in saffron. Mol. Biol. Res. Commun..

[45] Zhang F., Zhao J., Xu S., Fang W., Chen F., Teng N. (2017). MicroRNA and putative target discoveries in *Chrysanthemum* polyploidy breeding. Int. J. Genom..

[46] Yang J., Kulkarni K., Manolaridis I., Zhang Z., Dodd R.B., Mas-Droux C., Barford D. (2011). Mechanism of isoprenylcysteine carboxyl methylation from the crystal structure of the integral membrane methyltransferase ICMT. Mol. Cell.

[47] Gaynor M.L., Lim-Hing S., Mason C.M. (2020). Impact of genome duplication on secondary metabolite composition in non-cultivated species: A systematic meta-analysis. Ann. Bot. Lond..

[48] Bhuvaneswari G., Thirugnanasampandan R., Madhusudhanan G. (2020). Effect of colchicine induced tetraploidy on morphology, cytology, essential oil composition, gene expression and antioxidant activity of *Citrus limon* (L.) Osbeck. Physiol. Mol. Biol. Plants.

[49] Mansouri H., Bagheri M. (2017). Induction of polyploidy and its effect on *Cannabis sativa* L.. Cannabis Sativa L..

[50] Shmeit H., Fernández Y., Novy E., Kloucek P., Orosz P., Kokoska L. (2019). Autopolyploidy effect on morphological variation and essential oil in *Thymus vulgaris* L.. Sci. Hort..

[51] Iannicelli J., Elechosa M.A., Juárez M.A., Martínez A., Bugallo V., Bandoni A.L., Escandón A.S., van Baren C.M. (2016). Effect of polyploidization in the production of essential oils in *Lippia integrifolia*. Ind. Crop. Prod..

[52] Singh N.D., Kumar S., Daniell H. (2015). Expression of β-glucosidase increases trichome density and artemisinin content in transgenic *Artemisia annua* plants. Plant Biotechnol. J..

[53] Zhou F., Pichersky E. (2020). More is better: The diversity of terpene metabolism in plants. Curr. Opin. Plant Biol..

[54] Perl M. (2010). ATP synthesis and utilization in the early stage of seed germination in relation to seed dormancy and quality. Physiol. Plant..

[55] Caruso I., Lepore L., De Tommasi N., Piaz F.D., Frusciante L., Aversano R., Garramone R., Carputo D. (2011). Secondary metabolite profile in induced tetraploids of wild *Solanum commersonii* Dun. Chem. Biodivers.

[56] Aversano R., Scarano M.T., Aronne G., Caruso I., D’Amelia V., De Micco V., Fasano C., Termolino P., Carputo D. (2015). Genotype-specific changes associated to early synthesis of autotetraploids in wild potato species. Euphytica.

[57] Pai C.C., Kishkevich A., Deegan R.S., Keszthelyi A., Folkes L., Kearsey S.E., De Leon N., Soriano I., de Bruin R.A.M., Carr A.M. (2017). Set2 methyltransferase facilitates DNA replication and promotes genotoxic stress responses through MBF-dependent transcription. Cell Rep..

[58] Aguilera A., García-Muse T. (2013). Causes of genome instability. Annu. Rev. Genet..

[59] Murashige T., Skoog F. (1962). A Revised Medium for Rapid Growth and Bio Assays with Tobacco Tissue Cultures. Physiol. Plant..

[60] Bolger A.M., Lohse M., Usadel B. (2014). Trimmomatic: A flexible trimmer for Illumina sequence data. Bioinformatics.

[61] Bray N.L., Pimentel H., Melsted P., Pachter L. (2016). Near-optimal probabilistic RNA-seq quantification. Nat. Biotechnol..

[62] Du Z., Zhou X., Ling Y., Zhang Z.H., Su Z. (2010). AgriGO: A GO analysis toolkit for the agricultural community. Nucleic Acids Res..

[63] Supek F., Bosnjak M., Skunca N., Smuc T. (2011). REVIGO summarizes and visualizes long lists of gene ontology terms. PLoS ONE.

