# Supplementary material for: Whole-Genome Doubling Affects Pre-miRNA Expression in Plants
Source: Plants (Basel). 2021 May 18;10(5):1004. doi: 10.3390/plants10051004 (PMC8157229; doi:10.3390/plants10051004)
Supplement: Supplementary file 1 [file plants-10-01004-s001.zip › plants-1092947-supplementary.pdf]

Table S1. Number of common, 2x- and 4x-specific pre-miRNAs identified in each species studied here.

Species	Tissue	2x-specific	common 2x-4x	4x-specific
M. alba	leaves	2	30	6
A. thaliana	sepals	17	89	12
S. commersonii	leaves	11	118	46
B. rapa	floral buds	67	154	41
I. indigotica	leaves	18	56	11

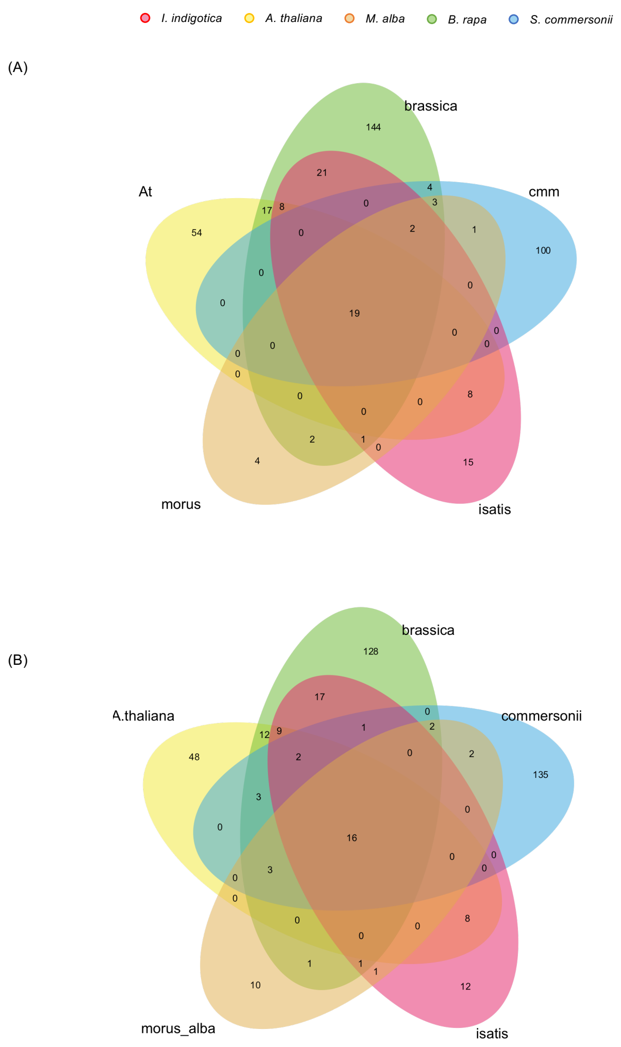
I. indigotica
A. thaliana
M. alba
B. rapa
S. commersonii
(A)
brassica
At
cmm
morus
isatis
144
21
4
3
17
8
0
54
0
2
1
100
0
0
0
19
0
0
0
0
0
0
0
0
0
8
0
0
2
1
0
4
15
(B)
brassica
A.thaliana
commersonii
morus_alba
isatis
128
17
0
12
9
1
2
2
48
2
0
135
3
0
0
16
0
0
3
0
0
0
0
8
0
0
1
1
1
10
12

Figure S1

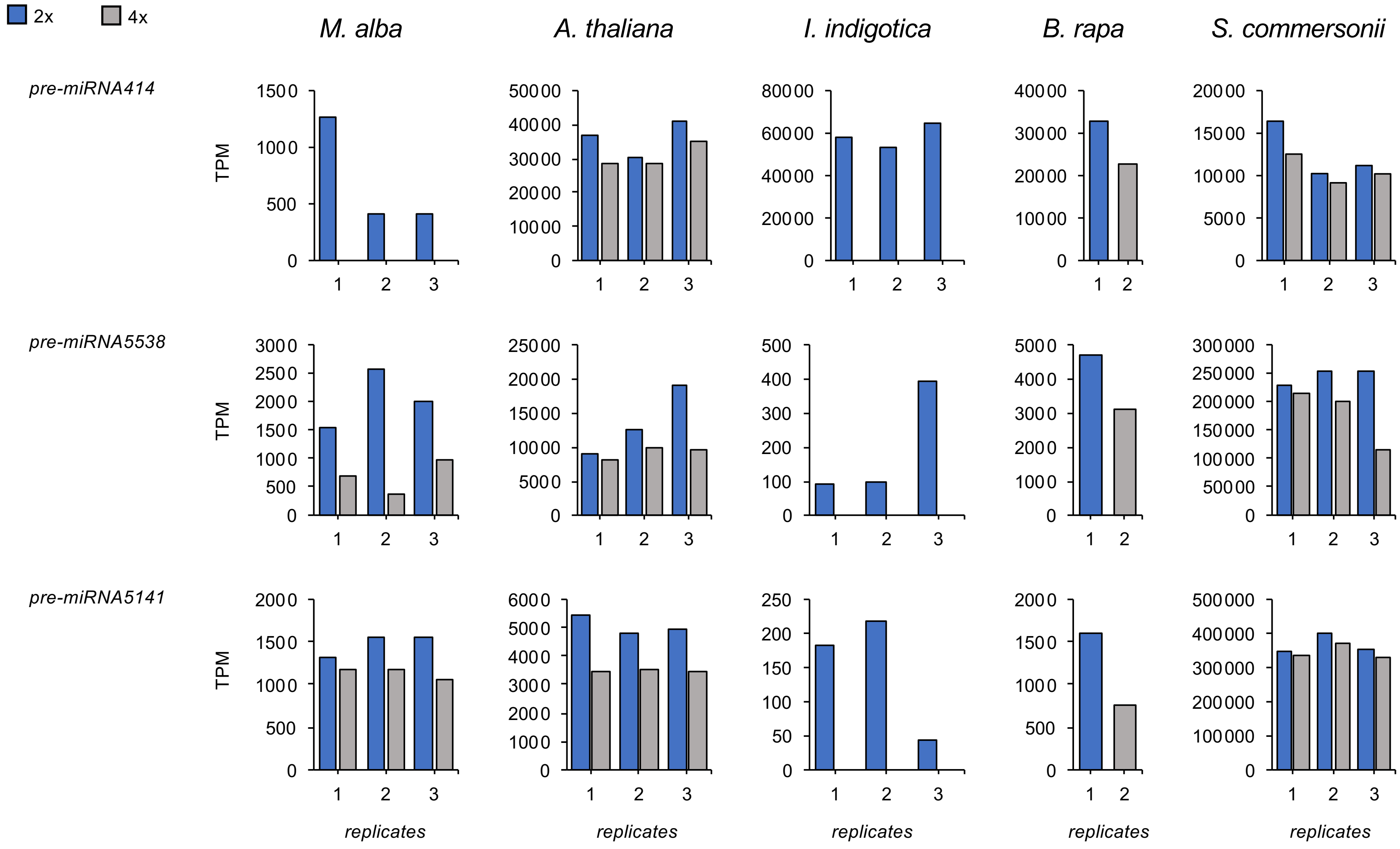
2x
4x
M. alba
A. thaliana
I. indigotica
B. rapa
S. commersonii
pre-miRNA414
pre-miRNA5538
pre-miRNA5141
TPM
replicates

Figure S2

A)

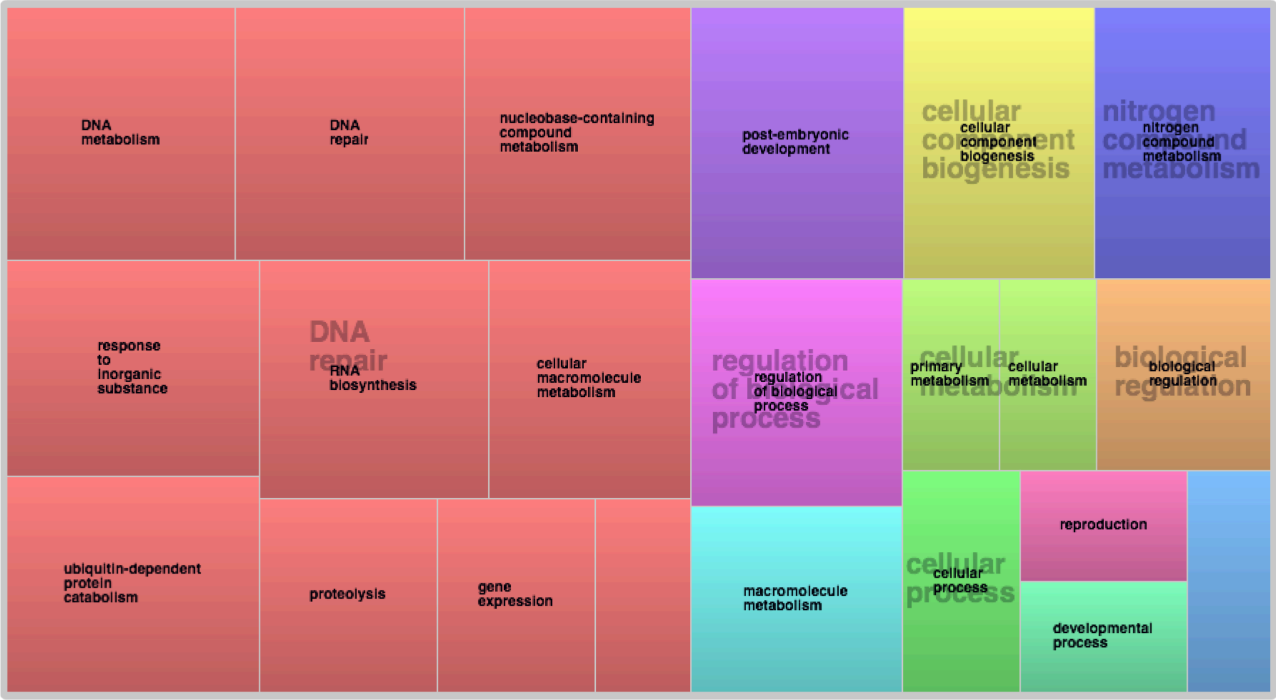

B)

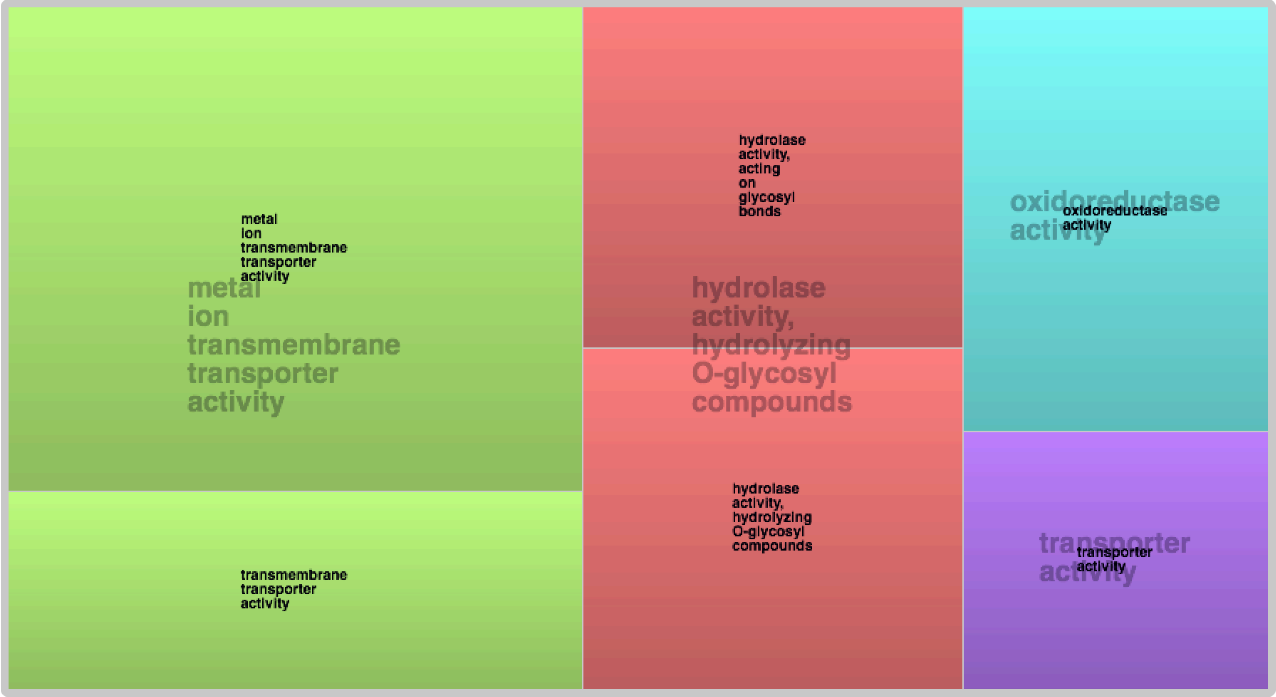

C)

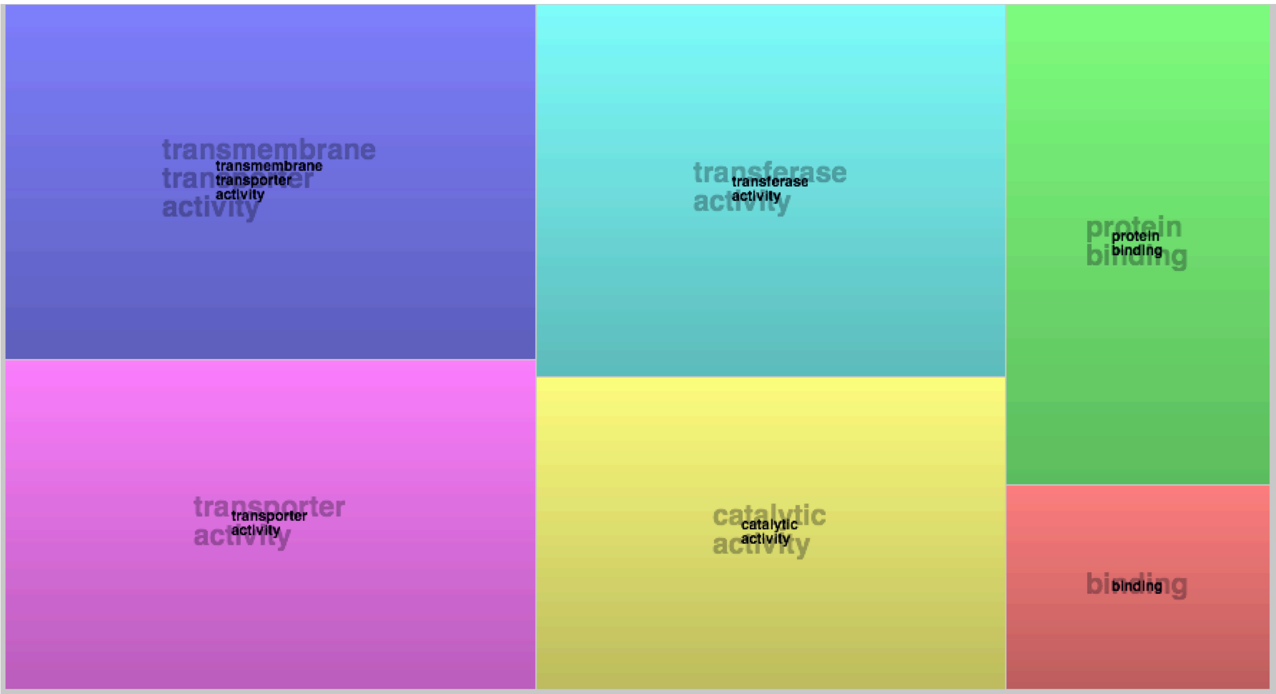

Figure S3

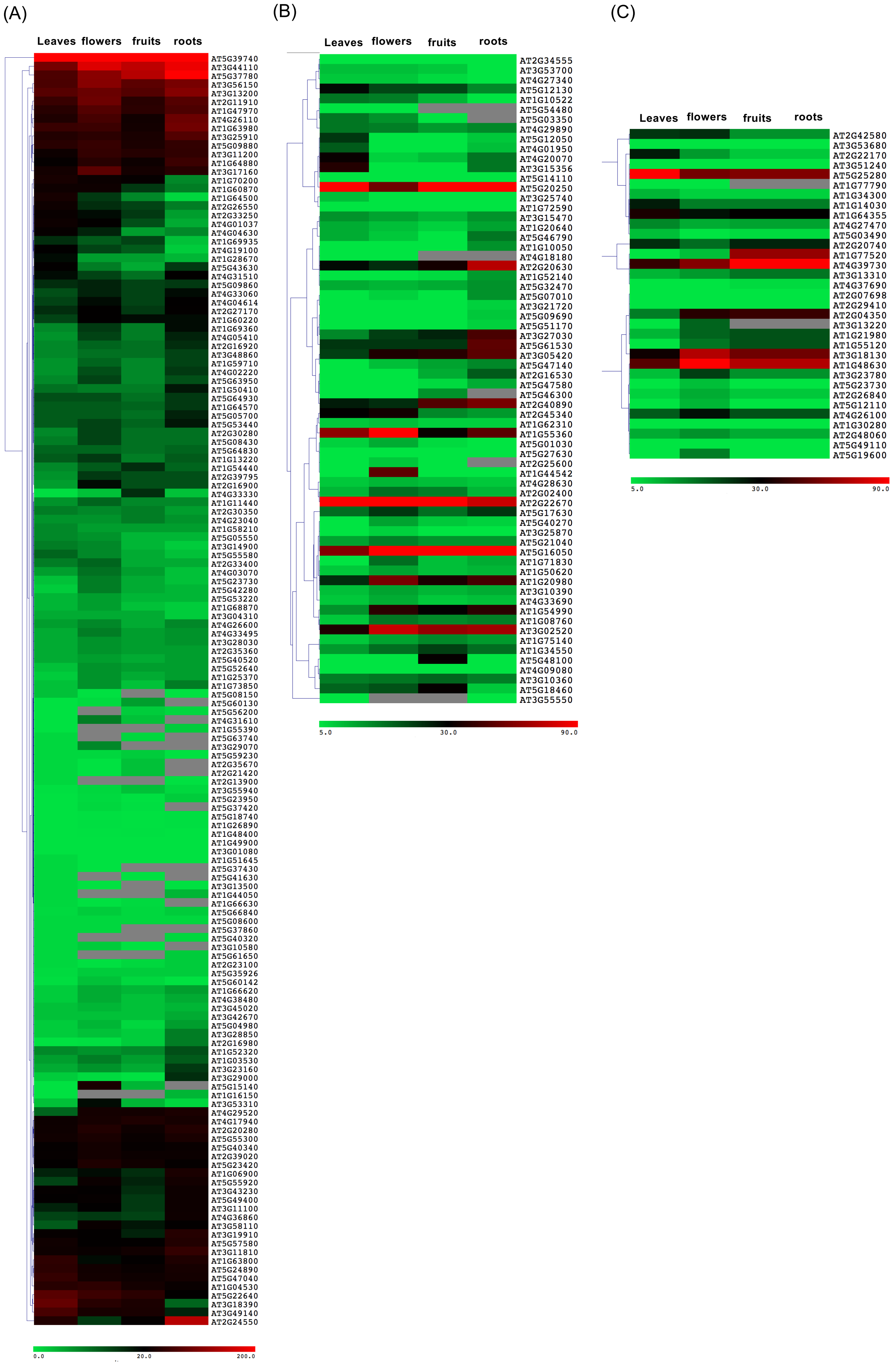
(A)
Leaves flowers fruits roots
AT5G39740
AT3G44110
AT5G37780
AT3G56150
AT3G13200
AT2G11910
AT1G47970
AT4G26110
AT1G63980
AT3G25910
AT5G09880
AT3G11200
AT1G64880
AT3G17160
AT1G70200
AT1G60870
AT1G64500
AT2G26550
AT2G33250
AT4G01037
AT4G04630
AT1G69935
AT4G19100
AT1G28670
AT5G43630
AT4G31510
AT5G09860
AT4G33060
AT4G04614
AT2G27170
AT1G60220
AT1G69360
AT4G05410
AT2G16920
AT3G48860
AT1G59710
AT4G02220
AT5G63950
AT1G50410
AT5G64930
AT1G64570
AT5G05700
AT5G53440
AT2G30280
AT5G08430
AT5G64830
AT1G13220
AT1G54440
AT2G39795
AT2G16900
AT4G33330
AT1G11440
AT2G30350
AT4G23040
AT1G58210
AT5G05550
AT3G14900
AT5G55580
AT2G33400
AT4G03070
AT5G23730
AT5G42280
AT5G53220
AT1G68870
AT3G04310
AT4G26600
AT4G33495
AT3G28030
AT2G35360
AT5G40520
AT5G52640
AT1G25370
AT1G73850
AT5G08150
AT5G60130
AT5G56200
AT4G31610
AT1G55390
AT5G63740
AT3G29070
AT5G59230
AT2G35670
AT2G21420
AT2G13900
AT3G55940
AT5G23950
AT5G37420
AT5G18740
AT1G26890
AT1G48400
AT1G49900
AT3G01080
AT1G51645
AT5G37430
AT5G41630
AT3G13500
AT1G44050
AT1G66630
AT5G66840
AT5G08600
AT5G37860
AT5G40320
AT3G10580
AT5G61650
AT2G23100
AT5G35926
AT5G60142
AT1G66620
AT4G38480
AT3G45020
AT3G42670
AT5G04980
AT3G28850
AT2G16980
AT1G52320
AT1G03530
AT3G23160
AT3G29000
AT5G15140
AT1G16150
AT3G53310
AT4G29520
AT4G17940
AT2G20280
AT5G55300
AT5G40340
AT2G39020
AT5G23420
AT1G06900
AT5G55920
AT3G43230
AT5G49400
AT3G11100
AT4G36860
AT3G58110
AT3G19910
AT5G57580
AT3G11810
AT1G63800
AT5G24890
AT5G47040
AT1G04530
AT5G22640
AT3G18390
AT3G49140
AT2G24550
0.0
20.0
200.0
(B)
Leaves flowers fruits roots
AT2G34555
AT3G53700
AT4G27340
AT5G12130
AT1G10522
AT5G54480
AT5G03350
AT4G29890
AT5G12050
AT4G01950
AT4G20070
AT3G15356
AT5G14110
AT5G20250
AT3G25740
AT1G72590
AT3G15470
AT1G20640
AT5G46790
AT1G10050
AT4G18180
AT2G20630
AT1G52140
AT5G32470
AT5G07010
AT3G21720
AT5G09690
AT5G51170
AT3G27030
AT5G61530
AT3G05420
AT5G47140
AT2G16530
AT5G47580
AT5G46300
AT2G40890
AT2G45340
AT1G62310
AT1G55360
AT5G01030
AT5G27630
AT2G25600
AT1G44542
AT4G28630
AT2G02400
AT2G22670
AT5G17630
AT5G40270
AT3G25870
AT5G21040
AT5G16050
AT1G71830
AT1G50620
AT1G20980
AT3G10390
AT4G33690
AT1G54990
AT1G08760
AT3G02520
AT1G75140
AT1G34550
AT5G48100
AT4G09080
AT3G10360
AT5G18460
AT3G55550
5.0
30.0
90.0
(C)
Leaves flowers fruits roots
AT2G42580
AT3G53680
AT2G22170
AT3G51240
AT5G25280
AT1G77790
AT1G34300
AT1G14030
AT1G64355
AT4G27470
AT5G03490
AT2G20740
AT1G77520
AT4G39730
AT3G13310
AT4G37690
AT2G07698
AT2G29410
AT2G04350
AT3G13220
AT1G21980
AT1G55120
AT3G18130
AT1G48630
AT3G23780
AT5G23730
AT2G26840
AT5G12110
AT4G26100
AT1G30280
AT2G48060
AT5G49110
AT5G19600
5.0
30.0
90.0

Figure S4

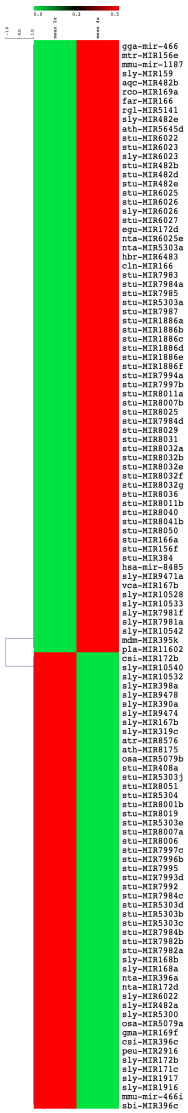
0.0
0.2
0.5
-1.0
0.0
1.0
mean 2x
mean 4x
gga-mir-466
mtr-MIR156e
mmu-mir-1187
sly-MIR159
aqc-MIR482b
rco-MIR169a
far-MIR166
rgl-MIR5141
sly-MIR482e
ath-MIR5645d
stu-MIR6022
stu-MIR6023
sly-MIR6023
stu-MIR482b
stu-MIR482d
stu-MIR482e
stu-MIR6025
stu-MIR6026
sly-MIR6026
stu-MIR6027
egu-MIR172d
nta-MIR6025e
nta-MIR5303a
hbr-MIR6483
cln-MIR166
stu-MIR7983
stu-MIR7984a
stu-MIR7985
stu-MIR5303a
stu-MIR7987
stu-MIR1886a
stu-MIR1886b
stu-MIR1886c
stu-MIR1886d
stu-MIR1886e
stu-MIR1886f
stu-MIR7994a
stu-MIR7997b
stu-MIR8011a
stu-MIR8007b
stu-MIR8025
stu-MIR7984d
stu-MIR8029
stu-MIR8031
stu-MIR8032a
stu-MIR8032b
stu-MIR8032e
stu-MIR8032f
stu-MIR8032g
stu-MIR8036
stu-MIR8011b
stu-MIR8040
stu-MIR8041b
stu-MIR8050
stu-MIR166a
stu-MIR156f
stu-MIR384
hsa-mir-8485
sly-MIR9471a
vca-MIR167b
sly-MIR10528
sly-MIR10533
sly-MIR7981f
sly-MIR7981a
sly-MIR10542
mdm-MIR395k
pla-MIR11602
csi-MIR172b
sly-MIR10540
sly-MIR10532
sly-MIR398a
sly-MIR9478
sly-MIR390a
sly-MIR9474
sly-MIR167b
sly-MIR319c
atr-MIR8576
ath-MIR8175
osa-MIR5079b
stu-MIR408a
stu-MIR5303j
stu-MIR8051
stu-MIR5304
stu-MIR8001b
stu-MIR8019
stu-MIR5303e
stu-MIR8007a
stu-MIR8006
stu-MIR7997c
stu-MIR7996b
stu-MIR7995
stu-MIR7993d
stu-MIR7992
stu-MIR7984c
stu-MIR5303d
stu-MIR5303b
stu-MIR5303c
stu-MIR7984b
stu-MIR7982b
stu-MIR7982a
sly-MIR168b
sly-MIR168a
nta-MIR396a
nta-MIR172d
sly-MIR6022
sly-MIR482a
sly-MIR5300
osa-MIR5079a
gma-MIR169f
csi-MIR396c
peu-MIR2916
sly-MIR172b
sly-MIR171c
sly-MIR1917
sly-MIR1916
mmu-mir-466i
sbi-MIR396c

Figure S5

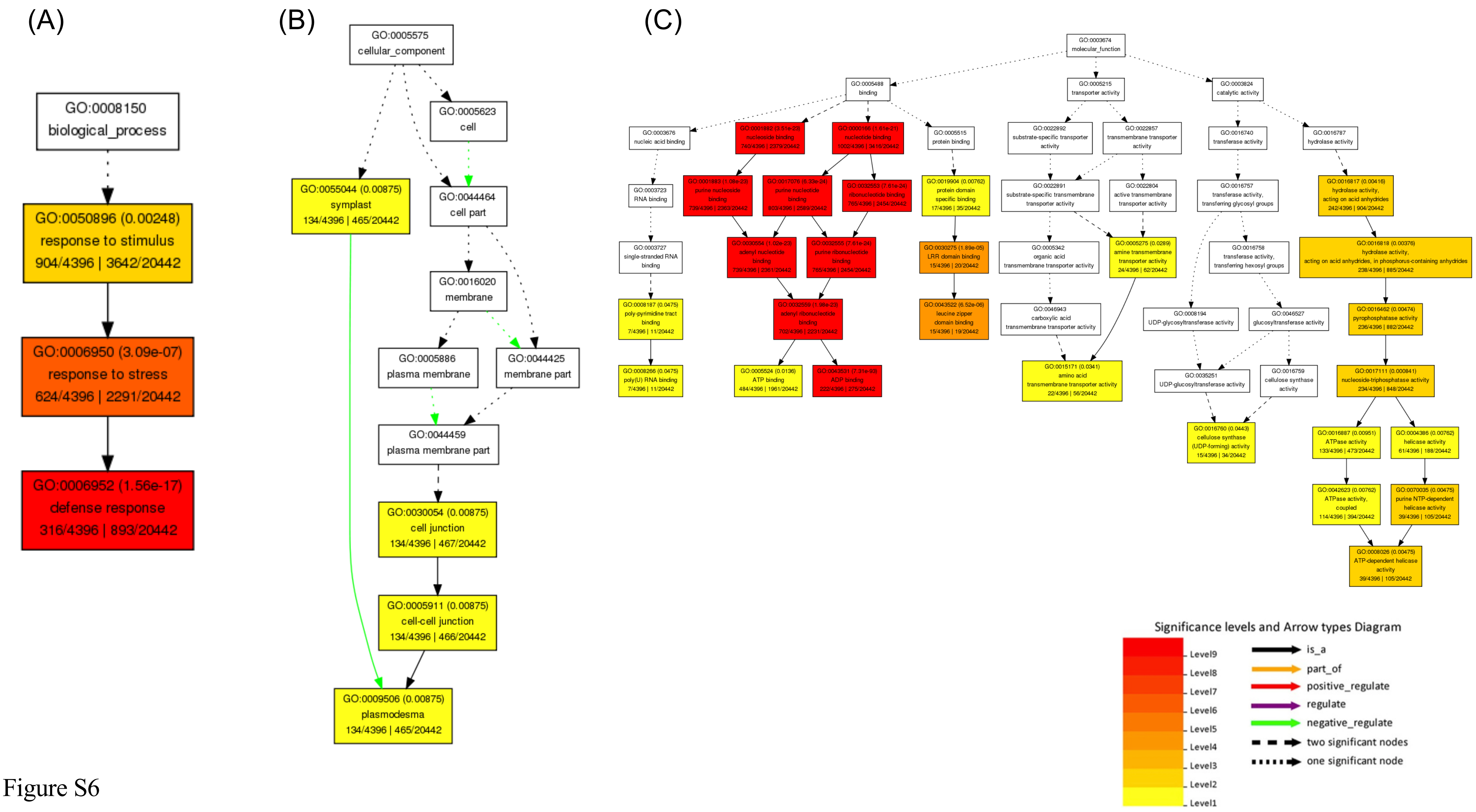
(A)
GO:0008150
biological_process
GO:0050896 (0.00248)
response to stimulus
904/4396 | 3642/20442
GO:0006950 (3.09e-07)
response to stress
624/4396 | 2291/20442
GO:0006952 (1.56e-17)
defense response
316/4396 | 893/20442
(B)
GO:0005575
cellular_component
GO:0005623
cell
GO:0055044 (0.00875)
symplast
134/4396 | 465/20442
GO:0044464
cell part
GO:0016020
membrane
GO:0005886
plasma membrane
GO:0044425
membrane part
GO:0044459
plasma membrane part
GO:0030054 (0.00875)
cell junction
134/4396 | 467/20442
GO:0005911 (0.00875)
cell-cell junction
134/4396 | 466/20442
GO:0009506 (0.00875)
plasmodesma
134/4396 | 465/20442
(C)
GO:0003674
molecular_function
GO:0005488
binding
GO:0005215
transporter activity
GO:0003824
catalytic activity
GO:0003676
nucleic acid binding
GO:0001882 (3.51e-23)
nucleoside binding
740/4396 | 2379/20442
GO:0000166 (1.61e-21)
nucleotide binding
1002/4396 | 3416/20442
GO:0005515
protein binding
GO:0022892
substrate-specific transporter activity
GO:0022857
transmembrane transporter activity
GO:0016740
transferase activity
GO:0016787
hydrolase activity
GO:0003723
RNA binding
GO:0001883 (1.08e-23)
purine nucleoside binding
739/4396 | 2363/20442
GO:0017076 (6.33e-24)
purine nucleotide binding
803/4396 | 2589/20442
GO:0032553 (7.61e-24)
ribonucleotide binding
765/4396 | 2454/20442
GO:0019904 (0.00762)
protein domain specific binding
17/4396 | 35/20442
GO:0022891
substrate-specific transmembrane transporter activity
GO:0022804
active transmembrane transporter activity
GO:0016757
transferase activity, transferring glycosyl groups
GO:0016817 (0.00416)
hydrolase activity, acting on acid anhydrides
242/4396 | 904/20442
GO:0003727
single-stranded RNA binding
GO:0030554 (1.02e-23)
adenyl nucleotide binding
739/4396 | 2361/20442
GO:0032555 (7.61e-24)
purine ribonucleotide binding
765/4396 | 2454/20442
GO:0030275 (1.89e-05)
LRR domain binding
15/4396 | 20/20442
GO:0005342
organic acid transmembrane transporter activity
GO:0005275 (0.0289)
amine transmembrane transporter activity
24/4396 | 62/20442
GO:0016758
transferase activity, transferring hexosyl groups
GO:0016818 (0.00376)
hydrolase activity, acting on acid anhydrides, in phosphorus-containing anhydrides
238/4396 | 885/20442
GO:0008187 (0.0475)
poly-pyrimidine tract binding
7/4396 | 11/20442
GO:0032559 (1.98e-23)
adenyl ribonucleotide binding
702/4396 | 2231/20442
GO:0043522 (6.52e-06)
leucine zipper domain binding
15/4396 | 19/20442
GO:0046943
carboxylic acid transmembrane transporter activity
GO:0008194
UDP-glycosyltransferase activity
GO:0046527
glucosyltransferase activity
GO:0016462 (0.00474)
pyrophosphatase activity
236/4396 | 882/20442
GO:0008266 (0.0475)
poly(U) RNA binding
7/4396 | 11/20442
GO:0005524 (0.0136)
ATP binding
484/4396 | 1961/20442
GO:0043531 (7.31e-93)
ADP binding
222/4396 | 275/20442
GO:0015171 (0.0341)
amino acid transmembrane transporter activity
22/4396 | 56/20442
GO:0035251
UDP-glucosyltransferase activity
GO:0016759
cellulose synthase activity
GO:0017111 (0.000841)
nucleoside-triphosphatase activity
234/4396 | 848/20442
GO:0016760 (0.0443)
cellulose synthase (UDP-forming) activity
15/4396 | 34/20442
GO:0016887 (0.00951)
ATPase activity
133/4396 | 473/20442
GO:0004386 (0.00762)
helicase activity
61/4396 | 188/20442
GO:0042623 (0.00762)
ATPase activity, coupled
114/4396 | 394/20442
GO:0070035 (0.00475)
purine NTP-dependent helicase activity
39/4396 | 105/20442
GO:0008026 (0.00475)
ATP-dependent helicase activity
39/4396 | 105/20442
Significance levels and Arrow types Diagram
Level9
Level8
Level7
Level6
Level5
Level4
Level3
Level2
Level1
is_a
part_of
positive_regulate
regulate
negative_regulate
two significant nodes
one significant node

Figure S6

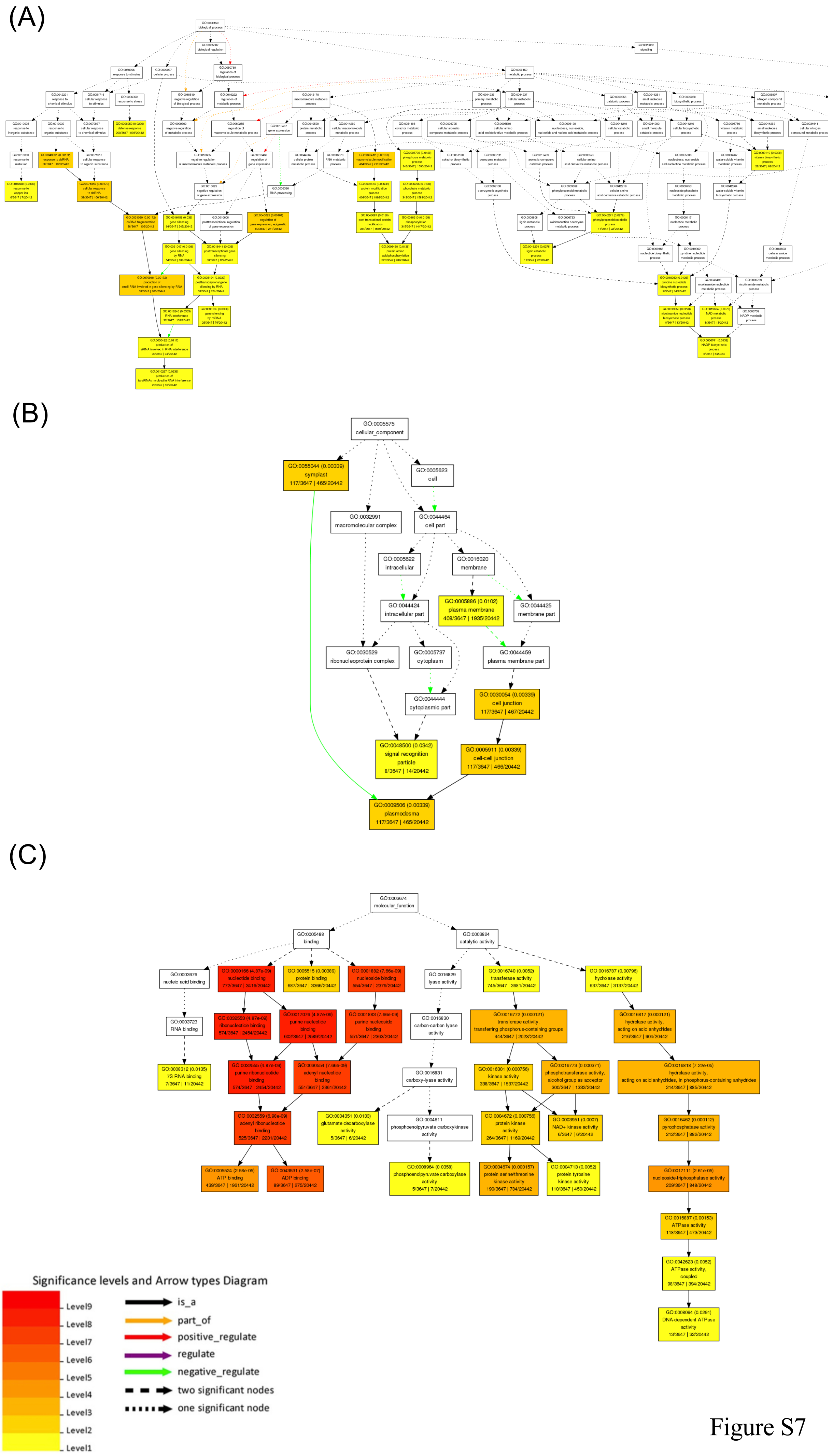
(A)
(B)
GO:0005575
cellular_component
GO:0055044 (0.00339)
symplast
117/3647 | 465/20442
GO:0005623
cell
GO:0032991
macromolecular complex
GO:0044464
cell part
GO:0005622
intracellular
GO:0016020
membrane
GO:0044424
intracellular part
GO:0005886 (0.0102)
plasma membrane
408/3647 | 1935/20442
GO:0044425
membrane part
GO:0030529
ribonucleoprotein complex
GO:0005737
cytoplasm
GO:0044459
plasma membrane part
GO:0044444
cytoplasmic part
GO:0030054 (0.00339)
cell junction
117/3647 | 467/20442
GO:0048500 (0.0342)
signal recognition
particle
8/3647 | 14/20442
GO:0005911 (0.00339)
cell-cell junction
117/3647 | 466/20442
GO:0009506 (0.00339)
plasmodesma
117/3647 | 465/20442
(C)
GO:0003674
molecular_function
GO:0005488
binding
GO:0003824
catalytic activity
GO:0003676
nucleic acid binding
GO:0000166 (4.87e-09)
nucleotide binding
772/3647 | 3416/20442
GO:0005515 (0.00389)
protein binding
687/3647 | 3366/20442
GO:0001882 (7.66e-09)
nucleoside binding
554/3647 | 2379/20442
GO:0016829
lyase activity
GO:0016740 (0.0052)
transferase activity
745/3647 | 3681/20442
GO:0016787 (0.00796)
hydrolase activity
637/3647 | 3137/20442
GO:0003723
RNA binding
GO:0032553 (4.87e-09)
ribonucleotide binding
574/3647 | 2454/20442
GO:0017076 (4.87e-09)
purine nucleotide
binding
602/3647 | 2589/20442
GO:0001883 (7.66e-09)
purine nucleoside
binding
551/3647 | 2363/20442
GO:0016830
carbon-carbon lyase
activity
GO:0016772 (0.000121)
transferase activity,
transferring phosphorus-containing groups
444/3647 | 2023/20442
GO:0016817 (0.000121)
hydrolase activity,
acting on acid anhydrides
216/3647 | 904/20442
GO:0008312 (0.0135)
7S RNA binding
7/3647 | 11/20442
GO:0032555 (4.87e-09)
purine ribonucleotide
binding
574/3647 | 2454/20442
GO:0030554 (7.66e-09)
adenyl nucleotide
binding
551/3647 | 2361/20442
GO:0016831
carboxy-lyase activity
GO:0016301 (0.000756)
kinase activity
338/3647 | 1537/20442
GO:0016773 (0.000371)
phosphotransferase activity,
alcohol group as acceptor
300/3647 | 1332/20442
GO:0016818 (7.22e-05)
hydrolase activity,
acting on acid anhydrides, in phosphorus-containing anhydrides
214/3647 | 885/20442
GO:0032559 (6.98e-09)
adenyl ribonucleotide
binding
525/3647 | 2231/20442
GO:0004351 (0.0133)
glutamate decarboxylase
activity
5/3647 | 6/20442
GO:0004611
phosphoenolpyruvate carboxykinase
activity
GO:0004672 (0.000756)
protein kinase
activity
264/3647 | 1169/20442
GO:0003951 (0.0007)
NAD+ kinase activity
6/3647 | 6/20442
GO:0016462 (0.000112)
pyrophosphatase activity
212/3647 | 882/20442
GO:0005524 (2.58e-05)
ATP binding
439/3647 | 1961/20442
GO:0043531 (2.58e-07)
ADP binding
89/3647 | 275/20442
GO:0008964 (0.0358)
phosphoenolpyruvate carboxylase
activity
5/3647 | 7/20442
GO:0004674 (0.000157)
protein serine/threonine
kinase activity
190/3647 | 784/20442
GO:0004713 (0.0052)
protein tyrosine
kinase activity
110/3647 | 450/20442
GO:0017111 (2.61e-05)
nucleoside-triphosphatase activity
209/3647 | 848/20442
GO:0016887 (0.00153)
ATPase activity
118/3647 | 473/20442
GO:0042623 (0.0052)
ATPase activity,
coupled
98/3647 | 394/20442
GO:0008094 (0.0291)
DNA-dependent ATPase
activity
13/3647 | 32/20442
Significance levels and Arrow types Diagram
Level9
Level8
Level7
Level6
Level5
Level4
Level3
Level2
Level1
is_a
part_of
positive_regulate
regulate
negative_regulate
two significant nodes
one significant node

Figure S7
